# Discovery of Akt Kinase Inhibitors through Structure-Based Virtual Screening and Their Evaluation as Potential Anticancer Agents

**DOI:** 10.3390/ijms16023202

**Published:** 2015-02-02

**Authors:** Chih-Hung Chuang, Ta-Chun Cheng, Yu-Ling Leu, Kuo-Hsiang Chuang, Shey-Cherng Tzou, Chien-Shu Chen

**Affiliations:** 1 Institutes of Basic Medical Sciences, National Cheng Kung University, Tainan 70101, Taiwan; E-Mail: a4132600@gmail.com; 2 Graduate Institute of Pharmacognosy, Taipei Medical University, Taipei 11031, Taiwan; E-Mails: chengtachun@gmail.com (T.-C.C.); khchuang@tmu.edu.tw (K.-H.C.); 3 Department of Pharmacy, Chia Nan University of Pharmacy and Science, Tainan 71710, Taiwan; E-Mail: wyulinlu@gmail.com; 4 Department of Biological Science and Technology, National Chiao Tung University, Hsinchu 30050, Taiwan; E-Mail: sctzou@gmail.com; 5 School of Pharmacy, China Medical University, Taichung 40402, Taiwan

**Keywords:** Akt kinase, inhibitors, cancer, virtual screening, docking

## Abstract

Akt acts as a pivotal regulator in the PI3K/Akt signaling pathway and represents a potential drug target for cancer therapy. To search for new inhibitors of Akt kinase, we performed a structure-based virtual screening using the DOCK 4.0 program and the X-ray crystal structure of human Akt kinase. From the virtual screening, 48 compounds were selected and subjected to the Akt kinase inhibition assay. Twenty-six of the test compounds showed more potent inhibitory effects on Akt kinase than the reference compound, H-89. These 26 compounds were further evaluated for their cytotoxicity against HCT-116 human colon cancer cells and HEK-293 normal human embryonic kidney cells. Twelve compounds were found to display more potent or comparable cytotoxic activity compared to compound H-89 against HCT-116 colon cancer cells. The best results were obtained with Compounds **a46** and **a48** having IC_50_ values (for HCT-116) of 11.1 and 9.5 µM, respectively, and selectivity indices (IC_50_ for HEK-293/IC_50_ for HCT-116) of 12.5 and 16.1, respectively. Through structure-based virtual screening and biological evaluations, we have successfully identified several new Akt inhibitors that displayed cytotoxic activity against HCT-116 human colon cancer cells. Especially, Compounds **a46** and **a48** may serve as useful lead compounds for further development of new anticancer agents.

## 1. Introduction

The phosphatidylinositol 3-kinase (PI3K)/Akt signaling pathway plays a critical role in regulating cell growth, survival, proliferation, protein synthesis and glycogen metabolism [1]. Deregulation of this pathway has been implicated in many human cancers. Thus, the PI3K/Akt pathway represents a potential target for cancer therapy [2]. Akt, also known as protein kinase B (PKB), is a serine/threonine protein kinase that acts as a pivotal regulator in the PI3K/Akt pathway. This kinase is activated by phosphorylation on Thr308 and Ser473. Once activated, Akt can phosphorylate a variety of downstream protein substrates, including GSK3β, BAD, FKHR and Mdm2. Akt is frequently amplified, overexpressed or overactivated in human cancer cells, including lung, breast, prostate, ovarian, gastric and pancreatic carcinomas [3,4,5]. Accordingly, inhibition of Akt is a promising therapeutic approach for the treatment of cancers [2,6].

**Figure 1:**
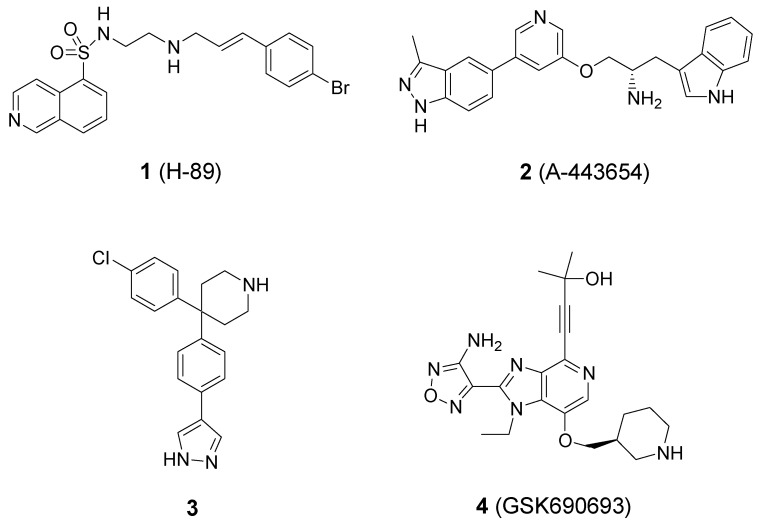
Representative ATP-competitive Akt kinase inhibitors **1**–**4**.

There are three known Akt isoforms: Akt1 (PKBα), Akt2 (PKBβ) and Akt3 (PKBγ) [7]. All three isoforms are composed of an *N*-terminal pleckstrin homology (PH) domain, a highly homologous kinase domain (sequence homology 90%–95%) and a *C*-terminal regulatory domain. The development of small-molecule Akt inhibitors is mainly focused on compounds that can bind to the ATP-binding site of kinase. To date, several classes of ATP-competitive Akt inhibitors have been reported, such as H-89 (**1**), A-443654 (**2**), pyrazole (**3**), and GSK690693 (**4**) (Figure 1) [8,9,10,11]. GSK690693 is a pan-Akt kinase inhibitor with low nanomolar activity [11]. This compound demonstrated *in vitro* and *in vivo* antiproliferative activity and could induce apoptosis *in vitro*. It also demonstrated antitumor activity in mice bearing human SKOV-3 ovarian, LNCaP prostate and BT474 and HCC-1954 breast carcinoma xenografts. GSK690693 is currently in a phase I clinical trial, given intravenously to patients with solid tumors or lymphoma. 

High-throughput screening (HTS) is generally used at present for lead compound generation. In recent years, as an alternative and complementary approach to HTS, virtual screening has emerged as a powerful, cost-effective and time-saving method for identifying lead compounds [12,13,14]. We are interested in identifying new Akt kinase inhibitors as potential anticancer agents by performing structure-based virtual screening using the DOCK program [15,16]. Once promising hit compounds are identified, they can serve as lead compounds for further optimization to develop new anticancer agents.

## 2. Results and Discussion

### 2.1. Computational Virtual Screening

To identify new Akt kinase inhibitors, structure-based virtual screening on a chemical database was performed using the program, DOCK (version 4.0). DOCK, one of the most widely-used docking programs, utilizes a sphere-matching algorithm to orient a putative ligand within the binding pocket of the protein. The DOCK program has been successfully applied to the discovery of biologically-active lead compounds in a number of diverse protein systems [17,18,19,20].

**Figure 2:**
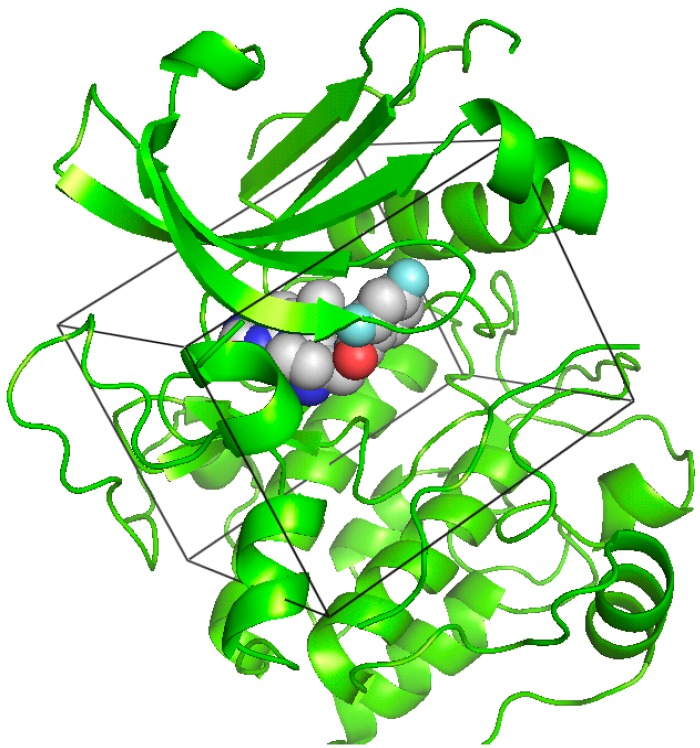
Crystal structure of the human Akt kinase domain in complex with a pyrrolopyrimidine inhibitor in the ATP-binding site (PDB Code 3MVH). A docking box was defined to enclose the ATP-binding site for virtual compound screening.

The virtual screening was performed using the X-ray crystal structure of human Akt in complex with a pyrrolopyrimidine inhibitor (Figure 2) retrieved from the Protein Data Bank (http://www.rcsb.org/pdb, PDB Code 3MVH) [21]. The chemical database for virtual screening was a subset of 35,367 compounds from the SPECS database. This database subset was built from the ZINC database website (http://zinc.docking.org) by extracting compounds (commercially available from the SPECS Company) that possess ring structures to potentially form hydrogen bonds with amino acid residues of a protein. The chemical database was computationally screened against the ATP-binding site of Akt using the DOCK program. The 1547 top-scoring compounds (energy score values ≤ −40.00 kcal/mol) were then visually inspected for the plausibility of their predicted binding modes to Akt kinase using the software, PyMOL.

Together with the consideration of the chemical diversity, the selection of compounds was assisted by the analysis of the docking models with respect to shape fitting, hydrogen-bonding and hydrophobic interactions. We finally selected and purchased 48 compounds for biological evaluation of Akt kinase inhibition. The ZINC codes and docking scores of 48 test compounds are listed in Table S1 in the Supplementary Material.

### 2.2. Akt Kinase Inhibition

**Figure 3:**
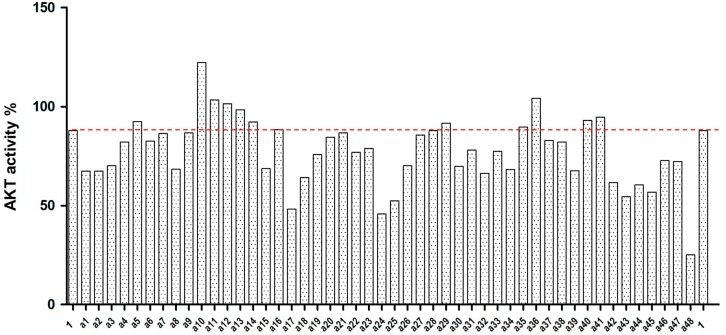
Results for Compounds **a1**–**a48** from the Akt kinase inhibition assay. H-89 (Compound **1**) was used as a reference inhibitor in the assay.

**Figure 4:**
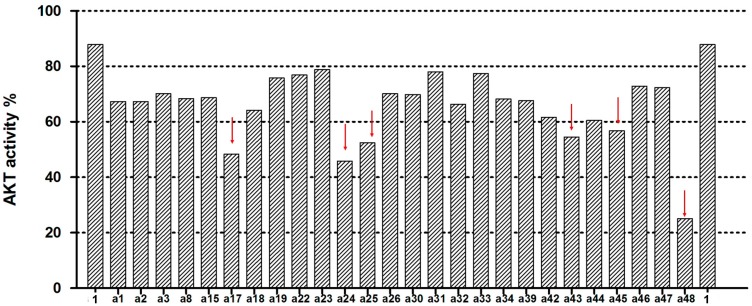
Twenty-six test compounds showed more potent inhibitory effects on Akt kinase than the reference inhibitor H-89 (Compound **1**). Six of these compounds inhibited Akt kinase to less than 60% residual activity (as indicated by the downward arrow).

The 48 selected compounds from virtual screening were assessed at 100 µM for their ability to inhibit Akt kinase activity using the EKS-400A assay kit (Enzo Life Sciences, Farmingdale, NY, USA). H-89 (Compound **1**) was used in the assay as a reference compound that can inhibit Akt kinase with an IC_50_ of 2.5 µM, as previously reported [8]. The results for Compounds **a1**–**a48** from the Akt kinase inhibition assay are shown in Figure 3. Twenty-six of the test compounds showed more potent inhibitory effects on Akt kinase than the reference compound, H-89 (Figure 4). Among them, six compounds (**a17**, **a24**, **a25**, **a43**, **a45** and **a48**) inhibited Akt kinase to less than 60% residual activity. In the present study, Compound **a48** was found to be the most active Akt inhibitor that exhibited 75% inhibition of Akt kinase activity at 100 µM. The IC_50_ values of Compounds **a46** and **a48** against Akt kinase were determined to be 201 and 158 µM, respectively. These results demonstrate our successful virtual screening approach to the discovery of new Akt kinase inhibitors.

### 2.3. Cytotoxicity Evaluation on HCT-116 Cancer Cells and HEK-293 Normal Cells

**Figure 5:**
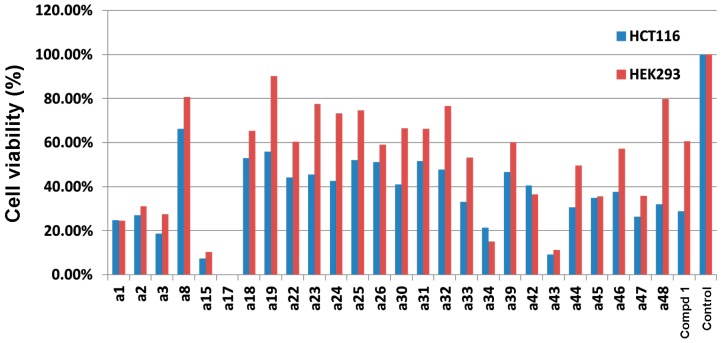
Results for 26 test compounds from cytotoxicity evaluation. Compounds were evaluated at 100 µM for their cytotoxicity against HCT-116 human colon cancer cells and HEK-293 normal human embryonic kidney cells. H-89 (Compound **1**) was used as a reference compound in the assays.

**Figure 6:**
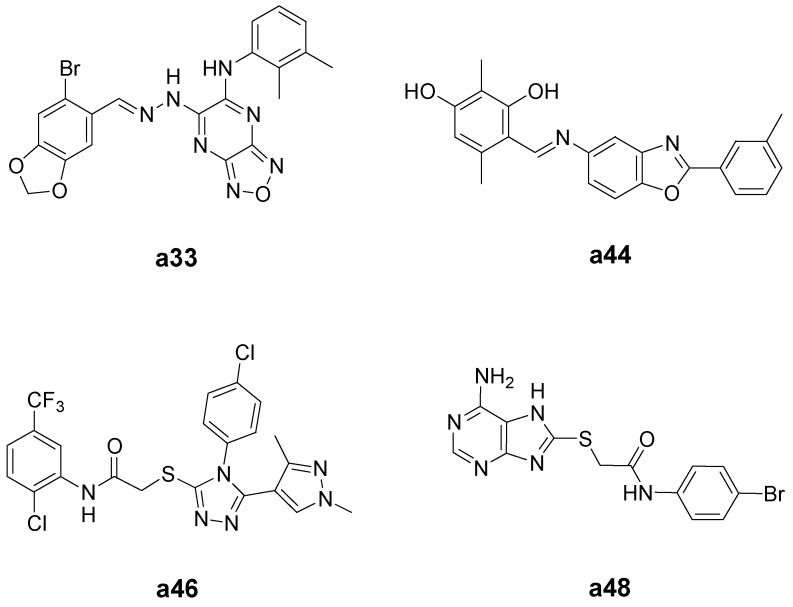
Chemical structures of Compounds **a33**, **a44**, **a46** and **a48**.

The 26 test compounds (as listed in Figure 4) were further evaluated at 100 µM for their cytotoxicity against HCT-116 human colon cancer cells and HEK-293 normal human embryonic kidney cells. H-89 (Compound **1**) was used as a reference compound in the assays. The results are shown in Figure 5. Twelve compounds (**a1**–**a3**, **a15**, **a17**, **a33**, **a34**, **a43**, **a44**, **a46**–**a48**) displayed more potent or comparable cytotoxic activity to that of H-89 against HCT-116 human colon cancer cells. Their chemical structures are shown in Figure S1 in the Supplementary Material. Taking into consideration the selectivity between cancer and normal cells, Compounds **a33**, **a44**, **a46** and **a48** (Figure 6) were selected for further determination of the IC_50_ values (Table 1). Lower IC_50_ values for HCT-116 colon cancer cells and better selectivity indices were obtained with Compounds **a44**, **a46** and **a48** than the reference compound, H-89. Especially, Compounds **a46** and **a48** showed promising results, having IC_50_ values (for HCT-116) of 11.1 and 9.5 µM, respectively, and selectivity indices (IC_50_ for HEK-293/IC_50_ for HCT-116) of 12.5 and 16.1, respectively. These two compounds may serve as lead compounds for further development of new anticancer agents. 

**Table 1:** Cytotoxicity (IC_50_ values) of Compounds **a33**, **a44**, **a46** and **a48** against human colon cancer cells (HCT-116) and normal human embryonic kidney cells (HEK-293).

Compound	IC_50_ (µM) ^a^		Selectivity index ^b^
	HCT-116	HEK-293	
**a33**	109.4	164.6	1.5
**a44**	50.6	101.5	2.0
**a46**	11.1	138.7	12.5
**a48**	9.5	152.6	16.1
H-89 ^c^	78.9	111.6	1.4

^a^ The concentration of compound required to inhibit cell growth by 50%; ^b^ the ratio of IC_50_ for HEK-293/IC_50_ for HCT-116; ^c^ H-89 (Compound **1**) was used as a reference compound in the assays.

### 2.4. Molecular Docking Studies

**Figure 7:**
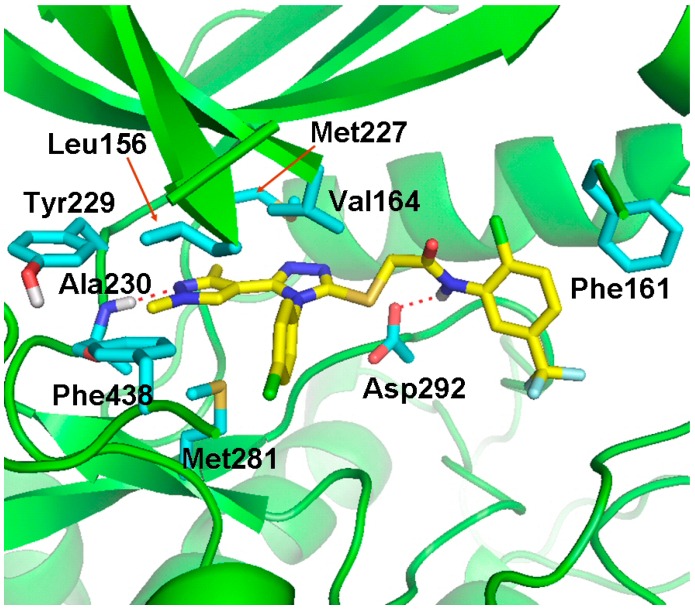
Docking model of Compound **a46** fit into the ATP-binding site of Akt kinase. Compound **a46** (yellow) and some representative amino acid residues (cyan) interacting with Compound **a46** are shown as stick structures. The red dashed lines indicate hydrogen-bonding interactions.

**Figure 8:**
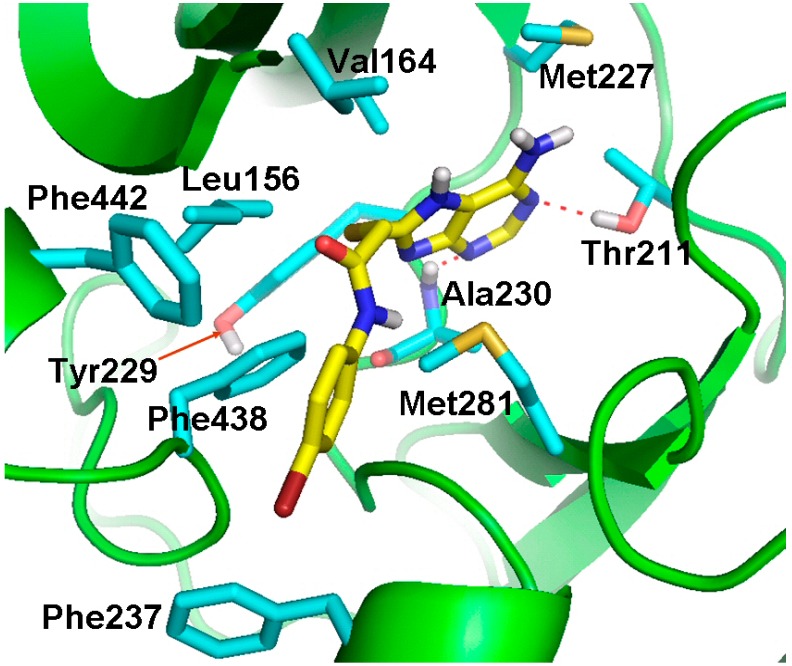
Docking model of Compound **a48** fit into the ATP-binding site of Akt kinase. Compound **a48** (yellow) and some representative amino acid residues (cyan) interacting with Compound **a48** are shown as stick structures. The red dashed lines indicate hydrogen-bonding interactions.

In the present study, Compounds **a46** and **a48** were shown to have the best results in the *in vitro* cytotoxicity evaluation. To predict the possible binding modes of Compounds **a46** and **a48** in the ATP-binding site of Akt kinase, we performed molecular docking studies using the docking program, GOLD 5.0 [22]. The GOLD program utilizes a genetic algorithm (GA) to perform flexible ligand docking simulations and, thus, may allow better prediction of the binding mode for a compound. The docking models for Compounds **a46** and **a48** are shown in Figure 7 and Figure 8, respectively. The predicted binding models indicate that there are favorable interactions, including hydrogen bonding and hydrophobic contacts between the inhibitor molecule and the Akt kinase. Compound **a46** forms hydrogen bonds with Ala230 and Asp292 and makes hydrophobic interactions with surrounding residues, including Leu156, Phe161, Val164, Met227, Tyr229, Met281 and Phe438. Compound **a48** is hydrogen-bonded to residues Thr211 and Ala230. This compound also has multiple hydrophobic interactions with surrounding residues, including Leu156, Val164, Met227, Tyr229, Phe237, Met281, Phe438 and Phe442.

## 3. Experimental Section 

### 3.1. Virtual Screening

The virtual screening was performed using the DOCK 4.0 program and the X-ray crystal structure of human Akt retrieved from the Protein Data Bank (http://www.rcsb.org/pdb, PDB Code 3MVH). The ATP-binding site of the Akt kinase domain was specified as the target site for ligand docking in virtual screening. Briefly, a molecular surface around the target site was generated with the MS program using a 1.4 Å probe radius, and this surface was used to generate, with the SPHGEN program, 60 overlapping spheres to fill the target site. A grid box enclosing the target site was created for grid calculations with dimensions of 22.8 × 25.9 × 19.8 Å. The force field scoring grids were calculated with the GRID program using a distance-dependent dielectric constant of 4*r*, an energy cutoff distance of 10 Å and a grid spacing of 0.3 Å. The database for virtual screening was a subset of 35,367 compounds from the SPECS database. This database subset was built from the ZINC database website by extracting compounds (available from the SPECS Company) with ring structures to potentially form hydrogen bonds with amino acid residues of a protein. The DOCK 4.0 program performs docking simulations using a distance-matching algorithm. The matching parameters used to run virtual screening were set as follows: distance tolerance = 0.5; distance minimum = 2.0; nodes maximum = 10; nodes minimum = 4; and critical points = yes. The chemical database was computationally screened against the ATP-binding site of the Akt kinase domain using the force field scoring function based on the interaction energy. Virtual screening was performed on a Silicon Graphics Octane workstation with dual 270-MHz MIPS R12000 processors.

For compound selection, the docking models of the 1547 top-ranked compounds (energy score values ≤ −40.00 kcal/mol) were visually inspected using the software, PyMOL. Together with the consideration of the chemical diversity, the selection of compounds was assisted by analysis of the docking models with respect to shape fitting, hydrogen-bonding and hydrophobic interactions. Finally, we selected 48 compounds for enzyme inhibition assays against Akt kinase. The compounds for testing were purchased from the SPECS Company.

### 3.2. Molecular Docking Studies

The X-ray crystal structure of human Akt kinase (PDB Code 3MVH) was used for docking studies of Compounds **a46** and **a48**. The small molecules and metal ions were removed, hydrogen atoms added and the resulting protein structure used in the docking simulation. The 3D structures of Compounds **a46** and **a48** were retrieved from the PubChem website (https://pubchem.ncbi.nlm.nih.gov/). Docking simulation was performed using the GOLD 5.0 program on an HP xw6600 workstation with Intel Xeon E5450/3.0 GHz Quadcores as the processors. The GOLD program utilizes a genetic algorithm (GA) to perform flexible ligand docking simulations. In the present study, for each of the 30 independent GA runs, a maximum number of 100,000 GA operations were performed on a single population of 100 individuals. Operator weights for crossover, mutation and migration were set to 95, 95 and 10, respectively. The GoldScore fitness function was applied for scoring the docking poses. The docking region was defined to encompass the ATP-binding site of Akt kinase domain. The best docking solution for a compound was chosen to represent the predicted binding mode to the ATP-binding site of Akt kinase.

### 3.3. Akt Kinase Inhibition Assay 

Compounds were evaluated at 100 µM for their ability to inhibit Akt kinase activity using the EKS-400A assay kit (Enzo Life Sciences). Test compounds or 1% DMSO (vehicle control) were incubated with the 8 ng pure Akt kinase in Hanks’ Balanced Salt Solution (HBSS) at 37 °C for 30 min. H-89 (Compound **1**) was used in the assay as a reference compound that can inhibit Akt kinase with an IC_50_ of 2.5 µM, as previously reported in the literature [8]. 

### 3.4. Cytotoxic Evaluation of Compounds

Compounds were evaluated at 100 µM for their cytotoxicity against HCT-116 human colon cancer cells and HEK-293 normal human embryonic kidney cells. Cells were seeded at a density of 1 × 10^4^ cells/well in 96-well plates and allowed to attach overnight. Cells were then treated with test compounds or 1% DMSO (vehicle control) and incubated at 37 °C in an atmosphere of 5% CO_2_ for 2 days. The ATPLite kit (PerkinElmer, Waltham, MA, USA) was used for detecting the cell viability.

## 4. Conclusions

Through structure-based virtual screening and biological evaluations, we have identified some new Akt kinase inhibitors that displayed cytotoxic activity against HCT-116 human colon cancer cells. Several of the new Akt inhibitors demonstrated more potent or comparable biological activities compared to the reference compound, H-89. Especially, Compounds **a46** and **a48** showed promising results, having IC_50_ values (for HCT-116) of 11.1 and 9.5 µM, respectively, and selectivity indices (IC_50_ for HEK-293/IC_50_ for HCT-116) of 12.5 and 16.1, respectively. Compounds **a46** and **a48** may serve as useful lead compounds for further development of new anticancer agents.

## Supplementary Materials

Supplementary materials can be found at http://www.mdpi.com/1422-0067/16/02/3202/s1.
